# Buffalo Water Supply

**Published:** 1903-08

**Authors:** 


### Buffalo Water Supply.

THE Buffalo pure water question has reached very nearly the acute stage, and after much discussion a joint hearing was held before the board of health and the committee on water of the Board of Aldermen, June 3, 1903.

It was thought best by those who had given the matter some thought that a commission composed of men acquainted with water conditions would be better able to clear up the situation and in time be competent to report back to the council the true status of affairs, such a commission to be composed of men who would devote time and energy to the study of the problem without hindrance from without or dissension from within. That is to say, it should be a capable, non-partisan and continuous body.

Moreover, such a commission could make a careful, comprehensive study of the water problem from the highest scientific standpoint; one which would stand the test of scientific criticism and yet adapt itself to practical and economical considerations. A commission thus organised could consider the water question from all possible standpoints, some of which we formulate:

1. Whether our city water is better or worse than the average of the large American cities.

2. Whether our city water is as impure as our city officials lead us to believe.

3. Whether better water could be secured in the lake or river other than at the present intake.

4. Whether better quality could be secured by pumping the water into reservoirs and, after sedimentation, allowing it to gravitate into the water mains.

5. Whether some means could not be devised to improve the water during the time of storms and upheavals on lake Erie.

6. Whether the contamination, if present, is due to local influences as Smokes Creek, the harbor, or back flow, or whether it is general and due to influences active at remote points.

The following observations appear germane to the issue :

1. If it should be found that our water supply is in good condition, then the water question should be dropped and our citizens saved from further anxiety and fear regarding its use.

2. If it should be found that our water supply is contaminated by local influences, then it should report the same to the proper authorities and suggest what steps should be taken to correct such pollution.

3. If the contamination is general and Lake Erie is found to be polluted from the sewer disposal and refuse of the communities bordering on its shores, then some steps should be taken to enlist the co-operation of the health boards of these cities with a view of preventing such pollution, or of asking the federal government to assist in this undertaking. Should it be found that our source of supply is contaminated and all efforts to correct the contamination prove unavailing, then this commission could study and report on the best method of filtration, leaving to the common council the question of ways and means for the construction and equipment of such a plant.

4. Such a commission should have the benefit of an expert in water analysis and an expert in sanitary science or a sanitary engineer, both to be the most practical and resourceful investigators that our country affords.

The common council has voted an appropriation of $2,000 for expert services, and some progress can now be made. A commission of five, however, patterned after the grade crossings commission—with power to secure experts—would have been more desirable, perhaps, than the plan adopted, of having the Board of Health, already overtaxed, do the work.

George W. Fuller, of New York, has been appointed by Commissioner Greene of the health department to examine the water supply. He will get $75 a day from the appropriation above referred to. In part, Mr. Fuller will deal with the question of establishing a water-filtration plant for the city.

---

The law providing for the State registration of nurses, passed by the last legislature, placed the appointment of the examining board in the hands of the regents of the University of the State of New York. From the candidates submitted by the executive committee of the New York State Nurses' Association, the regents have selected the following: For five years, Miss Jane Elizabeth Hitchcock, No. 265 Henry Street, New York. For four years, Miss Dorothy N. McDonald, No. 90 Hewes Street, Brooklyn. For three years, Miss Sophia F. Palmer, No. 149 Chestnut Street, Rochester. For two years, Miss Annie Damer, No. 76 Huron Street, Buffalo. For one year, L. Bissell Sanford, No. 217 East Twenty-seventh Street, New York.

Of these, Miss Hitchcock was graduated from the New York Hospital training school in 1891; Miss McDonald is a graduate of St. Mary's Hospital, Brooklyn, 1891; Miss Palmer is a graduate of the Massachusetts Hospital training school; Miss Damer is a graduate of Bellevue training school, 1886, and Mr. Sanford is a graduate of the Mills training school, Bellevue Hospital. The work of registration will now proceed as authorised by the law, the first meeting of the board having been fixed to occur in September next.

---

Messrs. Fairchild Brothers and Foster, of New York, are actively engaged in bringing to punishment all druggists who are guilty of substitution in any way that affects this celebrated house. The latest conviction is set forth in a decree of the special term of the supreme court of New York, made June 25, 1903, in favor of Fairchild Brothers and Foster, plaintiff, against James Kerr, defendant, which is as follows:

*Adjudged* that the defendant, his clerks, agents, servants and employes, be and they hereby are, enjoined and restrained perpetually from selling or dispensing either at the drug store of the said defendant, at West New Brighton, in the Borough of Richmond, of the City of New York, or elsewhere, any essence of pepsine, or pharmaceutical preparation of any sort or kind whatsoever, not manufactured by plaintiff, in imitation of, or in substitution for, Fairchild's essence of pepsine, whenever Fairchild's essence of pepsine is prescribed or asked for, and from representing by any word or action that any preparation sold by said defendant, not manufactured by plaintiff, is Fairchild's essence of pepsine, together with taxed costs.

If the Bostwick-Dowling drug substitution bill, which passed the legislature last winter, had become a law, it would have simplified these procedures very much indeed. It is to be hoped that before another year rolls around, the principles set forth in the bill above mentioned will be placed upon our statute books, as a part of the legal protection which should be accorded the citizen.

